# *N*-(4-Chloro­phen­yl)-1,1,1-trifluoro-*N*-(trifluoro­methyl­sulfon­yl)methane­sulfonamide

**DOI:** 10.1107/S1600536810003326

**Published:** 2010-02-03

**Authors:** Núbia Boechat, Adriana dos Santos Lages, Warner B. Kover, Edward R. T. Tiekink, James L. Wardell, Solange M. S. V. Wardell

**Affiliations:** a Fundação Oswaldo Cruz, Instituto de Tecnologia em Fármacos, Departamento de Síntese Orgânica, Manguinhos, CEP 21041250 Rio de Janeiro, RJ, Brazil; b Universidade Federal do Rio de Janeiro, Departamento de Química Orgânica, Instituto de Quıímica, Cidade Universitária, 21949-900 Rio de Janeiro, RJ, Brazil; c Department of Chemistry, University of Malaya, 50603 Kuala Lumpur, Malaysia; d Centro de Desenvolvimento Tecnológico em Saúde (CDTS), Fundação Oswaldo Cruz (FIOCRUZ), Casa Amarela, Campus de Manguinhos, Av. Brasil 4365, 21040-900 Rio de Janeiro, RJ, Brazil; e CHEMSOL, 1 Harcourt Road, Aberdeen AB15 5NY, Scotland

## Abstract

The title mol­ecule, also called 4-chloro-*N*,*N*-bis­(trifluoro­methane­sulfon­yl)aniline, C_8_H_4_ClF_6_NO_4_S_2_, has non-crystallographic twofold symmetry with the pseudo-axis aligned along the Cl—C⋯C—N backbone of the mol­ecule: the SO_2_CF_3_ residues lie to either side of the benzene ring. In the crystal, the presence of C—H⋯O contacts lead to the formation of a sequence of 12-membered {⋯HC_2_NSO}_2_ synthons within a supra­molecular chain aligned along [101].

## Related literature

For uses of *N*,*N*-bis­(trifluoro­methane­sulfon­yl)aniline derivatives, see: Zeller (2001 ▶); Wulff *et al.* (1986 ▶). For general background to the synthesis, see: Deprez *et al.* (1995 ▶); Greenfield & Crosanu (2008 ▶). For a previous synthesis of the title compound, see: Laali *et al.* (2007 ▶).
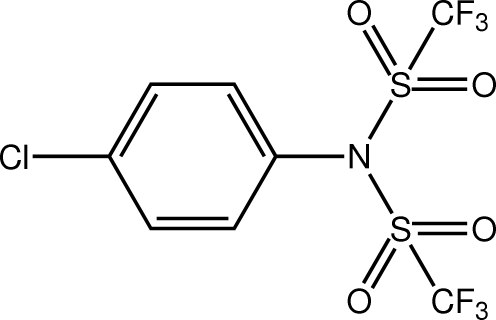


## Experimental

### 

#### Crystal data

C_8_H_4_ClF_6_NO_4_S_2_
*M* *_r_* = 391.70
Monoclinic, 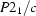

*a* = 11.5998 (3) Å
*b* = 13.4423 (4) Å
*c* = 9.0548 (2) Å
β = 108.014 (2)°
*V* = 1342.69 (6) Å^3^
*Z* = 4
Mo *K*α radiation
μ = 0.68 mm^−1^
*T* = 120 K
0.40 × 0.25 × 0.25 mm

#### Data collection

Nonius KappaCCD area-detector diffractometer
Absorption correction: multi-scan (*SADABS*; Sheldrick, 2007 ▶) *T*_min_ = 0.821, *T*_max_ = 1.000
17100 measured reflections
3047 independent reflections
2806 reflections with *I* > 2σ(*I*)
*R*_int_ = 0.023

#### Refinement

*R*[*F* ^2^ > 2σ(*F* ^2^)] = 0.031
*wR*(*F* ^2^) = 0.082
*S* = 1.04
3047 reflections
199 parameters
H-atom parameters constrained
Δρ_max_ = 0.62 e Å^−3^
Δρ_min_ = −0.41 e Å^−3^

### 

Data collection: *COLLECT* (Hooft, 1998 ▶); cell refinement: *DENZO* (Otwinowski & Minor, 1997 ▶) and *COLLECT*; data reduction: *DENZO* and *COLLECT*; program(s) used to solve structure: *SHELXS97* (Sheldrick, 2008 ▶); program(s) used to refine structure: *SHELXL97* (Sheldrick, 2008 ▶); molecular graphics: *ORTEP-3* (Farrugia, 1997 ▶) and *DIAMOND* (Brandenburg, 2006 ▶); software used to prepare material for publication: *publCIF* (Westrip, 2010 ▶).

## Supplementary Material

Crystal structure: contains datablocks global, I. DOI: 10.1107/S1600536810003326/hb5319sup1.cif

Structure factors: contains datablocks I. DOI: 10.1107/S1600536810003326/hb5319Isup2.hkl

Additional supplementary materials:  crystallographic information; 3D view; checkCIF report

## Figures and Tables

**Table 1:** Hydrogen-bond geometry (Å, °)

*D*—H⋯*A*	*D*—H	H⋯*A*	*D*⋯*A*	*D*—H⋯*A*
C3—H3⋯O3^i^	0.95	2.57	3.496 (2)	164
C5—H5⋯O1^ii^	0.95	2.59	3.385 (2)	141

Symmetry codes: (i) 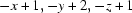; (ii) 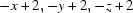.

**Fig. 1.**
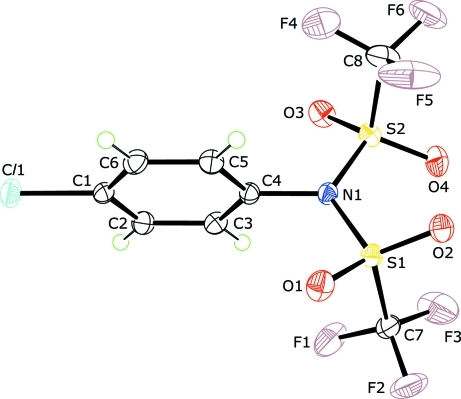
The molecular structure of (I) showing displacement ellipsoids at the 50% probability level.

**Fig. 2.**
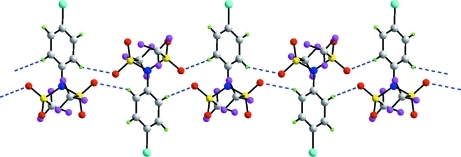
A view of the supramolecular chain in (I) aligned along [1 0 1] and mediated by C–H···O interactions (blue dashed lines). Colour code: Cl, cyan; S, yellow; F, pink; O, red; N, blue; C, grey; and H, green.

**Fig. 3.**
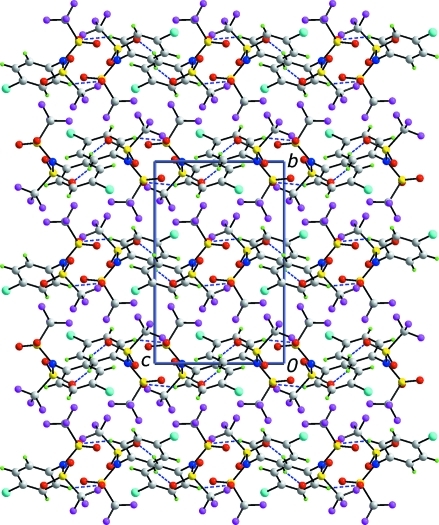
View of the stacking of layers in (I). The C–H···O interactions are shown as blue dashed lines. Colour code: Cl, cyan; S, yellow; F, pink; O, red; N, blue; C, grey; and H, green.

## supplementary crystallographic information

### Comment

*N*,*N*-Bis(trifluoromethanesulfonyl)aniline derivatives find use in synthetic chemistry such as mild triflating reagents (Zeller, 2001; Wulff *et al.*, 1986). Following a literature procedure to *p*-ClC_6_H_4_NHSO_2_CF_3_, using *p*-ClC_6_H_4_NH_2_, (F_3_CSO_2_)O and Et_3_N in CH_2_Cl_2_ at 213 K, a little of the di-substituted compound, *p*-ClC_6_H_4_(NSO_2_CF_3_), (I), was isolated as a side-product (Greenfield & Crosanu, 2008; Deprez *et al.*, 1995). Compound (I) has been reported previously (Laali *et al.*, 2007) and the X-ray structure determination is reported herein.

In (I), the SO_2_CF_3_ groups occupy approximately orthogonal positions to either side of the aromatic ring: the C3/C4/N1/S1, S2 torsion angles are 77.44 (17) and -101.55 (15) °, respectively; the dihedral angle formed between the benzene ring and NS_2_ group is 78.13 (6) °. The CF_3_ groups lie to either side of the molecule and fold back over the benzene ring so that, overall, the molecule has non-crystallographic 2-fold symmetry when viewed down the Cl1–C1–C4–N1 axis.

Supramolecular aggregation in (I) is dominated by C–H···O interactions that lead to the formation of a sequence of 12-membered {···HC_2_NSO}_2_ synthons aligned along [1 0 1], Fig. 2 and Table 1. Chains are connected into layers through the agency of C–Cl···π interactions between centrosymmetrically related residues [C1–Cl1···ring centroid(C1–C6)^i^ = 3.4592 (8) Å with angle at Cl1 = 92.46 (6) ° for i: 2-*x*, 2-*y*, 1-*z*]. The layers thus formed stack along the *b* axis with the closest contacts between successive layers being of the type C–F···π [C8–F4···ring centroid(C1–C6)^ii^ = 3.4708 (16) Å with angle at F4 = 122.83 (11) ° for *ii*: *x*, 3/2-*y*,1/2+*z*].

### Experimental

To a cooled (213 K) solution of *p*-ClC_6_H_4_NH_2_ (11.5 g, 9.0 mmol) and triethylamine (1.50 ml; 10.8 mmol, 1.20 eq.) in CH_2_Cl_2_ (40 ml) was slowly added a solution of trifluoromethanesulfonic anhydride (2.40 ml; 13.5 mmol, 1.50 eq) in CH_2_Cl_2_ (40 ml). After the mixture was stirred at 213-223 K for 1 h, water (30 ml) was added. The mixture was allowed to warm to room temperature, and the organic layer was decanted, washed with water, dried, and evaporated. The products, *N*-(4-chlorophenyl)trifluoromethylsulfonamide and *N*-(4-chlorophenyl)-bis-trifluoromethylsulfonamide, (I), were purified by chromatography on silica gel with hexane as eluent. Products were recrystallized from hexane. Characterisation data for (I): m.pt. 346-348 K, ^1^H-NMR (500 MHz, CDCl_3_) δ: 7.34 (2*H*), 7.50 (2*H*) p.p.m. ^13^C-NMR (125 MHz, CDCl_3_) δ: 119.36 (q, ^1^J(C,F) = 325 Hz), 125.17 (C3), 130.06, 132.18, 138.84 p.p.m. ^19^F-NMR (376 MHz, CDCl_3_) δ: 71.11 p.p.m.

### Refinement

The C-bound H atoms were geometrically placed (C–H = 0.95 Å) and refined as riding with U_iso_(H) = 1.2U_eq_(C).

### Crystal data

**Table:**

C_8_H_4_ClF_6_NO_4_S_2_	*F*(000) = 776
*M**_r_* = 391.70	*D*_x_ = 1.938 Mg m^−^^3^
Monoclinic, *P*2_1_/*c*	Mo *K*α radiation, λ = 0.71073 Å
Hall symbol: -P 2ybc	Cell parameters from 2983 reflections
*a* = 11.5998 (3) Å	θ = 2.9–27.5°
*b* = 13.4423 (4) Å	µ = 0.68 mm^−^^1^
*c* = 9.0548 (2) Å	*T* = 120 K
β = 108.014 (2)°	Block, colourless
*V* = 1342.69 (6) Å^3^	0.40 × 0.25 × 0.25 mm
*Z* = 4	

### Data collection

**Table:**

Nonius KappaCCD area-detector diffractometer	3047 independent reflections
Radiation source: Enraf Nonius FR591 rotating anode	2806 reflections with *I* > 2σ(*I*)
10 cm confocal mirrors	*R*_int_ = 0.023
Detector resolution: 9.091 pixels mm^-1^	θ_max_ = 27.5°, θ_min_ = 2.9°
φ and ω scans	*h* = −15→15
Absorption correction: multi-scan (*SADABS*; Sheldrick, 2007)	*k* = −17→17
*T*_min_ = 0.821, *T*_max_ = 1.000	*l* = −11→11
17100 measured reflections	

### Refinement

**Table:**

Refinement on *F*^2^	Primary atom site location: structure-invariant direct methods
Least-squares matrix: full	Secondary atom site location: difference Fourier map
*R*[*F*^2^ > 2σ(*F*^2^)] = 0.031	Hydrogen site location: inferred from neighbouring sites
*wR*(*F*^2^) = 0.082	H-atom parameters constrained
*S* = 1.04	*w* = 1/[σ^2^(*F*_o_^2^) + (0.0407*P*)^2^ + 1.1662*P*] where *P* = (*F*_o_^2^ + 2*F*_c_^2^)/3
3047 reflections	(Δ/σ)_max_ = 0.001
199 parameters	Δρ_max_ = 0.62 e Å^−^^3^
0 restraints	Δρ_min_ = −0.41 e Å^−^^3^

### Special details

**Table:**

Geometry. All s.u.'s (except the s.u. in the dihedral angle between two l.s. planes) are estimated using the full covariance matrix. The cell s.u.'s are taken into account individually in the estimation of s.u.'s in distances, angles and torsion angles; correlations between s.u.'s in cell parameters are only used when they are defined by crystal symmetry. An approximate (isotropic) treatment of cell s.u.'s is used for estimating s.u.'s involving l.s. planes.
Refinement. Refinement of *F*^2^ against ALL reflections. The weighted *R*-factor wR and goodness of fit *S* are based on *F*^2^, conventional *R*-factors *R* are based on *F*, with *F* set to zero for negative *F*^2^. The threshold expression of *F*^2^ > 2σ(*F*^2^) is used only for calculating *R*-factors(gt) etc. and is not relevant to the choice of reflections for refinement. *R*-factors based on *F*^2^ are statistically about twice as large as those based on *F*, and *R*- factors based on ALL data will be even larger.

### Fractional atomic coordinates and isotropic or equivalent isotropic displacement parameters (Å^2^)

**Table:**

	*x*	*y*	*z*	*U*_iso_*/*U*_eq_	
Cl1	0.97286 (4)	0.86092 (3)	0.34987 (5)	0.02733 (12)	
S1	0.78053 (4)	1.09197 (3)	0.91222 (4)	0.01889 (11)	
S2	0.59031 (4)	0.94255 (3)	0.78741 (5)	0.01943 (11)	
F1	0.70006 (16)	1.20000 (9)	0.66292 (14)	0.0497 (4)	
F2	0.75847 (13)	1.28200 (9)	0.87505 (16)	0.0449 (3)	
F3	0.58975 (12)	1.20628 (11)	0.8139 (2)	0.0551 (4)	
F4	0.69058 (14)	0.77016 (10)	0.86879 (18)	0.0525 (4)	
F5	0.71980 (16)	0.87281 (11)	1.05802 (17)	0.0663 (5)	
F6	0.54674 (13)	0.80495 (10)	0.96076 (16)	0.0478 (3)	
O1	0.90194 (11)	1.10259 (10)	0.91208 (15)	0.0280 (3)	
O2	0.74816 (13)	1.08338 (10)	1.04986 (15)	0.0293 (3)	
O3	0.54372 (11)	0.89593 (10)	0.64061 (14)	0.0254 (3)	
O4	0.52094 (12)	1.00882 (11)	0.84680 (16)	0.0308 (3)	
N1	0.72049 (12)	0.99798 (10)	0.79108 (15)	0.0167 (3)	
C1	0.89885 (15)	0.90047 (12)	0.4795 (2)	0.0192 (3)	
C2	0.79853 (15)	0.96166 (12)	0.42518 (19)	0.0201 (3)	
H2	0.7706	0.9812	0.3192	0.024*	
C3	0.73954 (14)	0.99385 (12)	0.52852 (18)	0.0182 (3)	
H3	0.6704	1.0358	0.4943	0.022*	
C4	0.78292 (14)	0.96396 (12)	0.68241 (18)	0.0162 (3)	
C5	0.88287 (15)	0.90243 (12)	0.73621 (19)	0.0198 (3)	
H5	0.9106	0.8824	0.8420	0.024*	
C6	0.94185 (15)	0.87055 (12)	0.6327 (2)	0.0218 (3)	
H6	1.0110	0.8286	0.6668	0.026*	
C7	0.70024 (18)	1.20199 (13)	0.8072 (2)	0.0258 (4)	
C8	0.64203 (18)	0.84073 (14)	0.9298 (2)	0.0301 (4)	

### Atomic displacement parameters (Å^2^)

**Table:**

	*U*^11^	*U*^22^	*U*^33^	*U*^12^	*U*^13^	*U*^23^
Cl1	0.0291 (2)	0.0285 (2)	0.0294 (2)	−0.00347 (16)	0.01644 (18)	−0.01089 (17)
S1	0.0231 (2)	0.0187 (2)	0.01481 (19)	−0.00119 (14)	0.00583 (15)	−0.00231 (13)
S2	0.0178 (2)	0.0220 (2)	0.0187 (2)	−0.00101 (14)	0.00585 (15)	0.00126 (14)
F1	0.0996 (12)	0.0286 (6)	0.0236 (6)	0.0191 (7)	0.0229 (7)	0.0060 (5)
F2	0.0691 (9)	0.0188 (5)	0.0461 (7)	−0.0062 (6)	0.0167 (7)	−0.0067 (5)
F3	0.0402 (7)	0.0402 (7)	0.0918 (12)	0.0177 (6)	0.0303 (8)	0.0267 (7)
F4	0.0650 (9)	0.0347 (7)	0.0620 (9)	0.0206 (7)	0.0257 (8)	0.0194 (6)
F5	0.0872 (11)	0.0427 (8)	0.0367 (7)	−0.0277 (8)	−0.0281 (7)	0.0189 (6)
F6	0.0593 (9)	0.0433 (7)	0.0469 (8)	−0.0179 (6)	0.0252 (7)	0.0100 (6)
O1	0.0216 (6)	0.0317 (7)	0.0290 (7)	−0.0046 (5)	0.0053 (5)	−0.0099 (5)
O2	0.0462 (8)	0.0276 (7)	0.0174 (6)	−0.0026 (6)	0.0146 (6)	−0.0025 (5)
O3	0.0211 (6)	0.0313 (7)	0.0216 (6)	−0.0065 (5)	0.0032 (5)	−0.0025 (5)
O4	0.0267 (7)	0.0358 (7)	0.0357 (7)	0.0025 (6)	0.0182 (6)	−0.0020 (6)
N1	0.0178 (6)	0.0171 (6)	0.0158 (6)	−0.0006 (5)	0.0060 (5)	−0.0025 (5)
C1	0.0216 (8)	0.0159 (7)	0.0226 (8)	−0.0044 (6)	0.0105 (6)	−0.0059 (6)
C2	0.0241 (8)	0.0204 (8)	0.0152 (7)	−0.0020 (6)	0.0055 (6)	−0.0020 (6)
C3	0.0182 (7)	0.0175 (7)	0.0171 (7)	0.0018 (6)	0.0027 (6)	0.0002 (6)
C4	0.0163 (7)	0.0166 (7)	0.0160 (7)	−0.0004 (6)	0.0055 (6)	−0.0015 (6)
C5	0.0197 (8)	0.0207 (8)	0.0176 (8)	0.0021 (6)	0.0038 (6)	0.0024 (6)
C6	0.0190 (8)	0.0193 (8)	0.0269 (8)	0.0029 (6)	0.0069 (7)	−0.0003 (6)
C7	0.0371 (10)	0.0185 (8)	0.0244 (9)	0.0017 (7)	0.0134 (7)	0.0002 (6)
C8	0.0360 (10)	0.0247 (9)	0.0258 (9)	−0.0082 (7)	0.0040 (8)	0.0053 (7)

### Geometric parameters (Å, °)

**Table:**

Cl1—C1	1.7380 (16)	F5—C8	1.304 (2)
S1—O2	1.4131 (13)	F6—C8	1.313 (2)
S1—O1	1.4160 (13)	N1—C4	1.4637 (19)
S1—N1	1.6769 (13)	C1—C6	1.381 (2)
S1—C7	1.8470 (18)	C1—C2	1.385 (2)
S2—O4	1.4135 (13)	C2—C3	1.388 (2)
S2—O3	1.4168 (13)	C2—H2	0.9500
S2—N1	1.6749 (14)	C3—C4	1.387 (2)
S2—C8	1.8483 (19)	C3—H3	0.9500
F1—C7	1.306 (2)	C4—C5	1.384 (2)
F2—C7	1.317 (2)	C5—C6	1.388 (2)
F3—C7	1.303 (2)	C5—H5	0.9500
F4—C8	1.309 (3)	C6—H6	0.9500
			
O2—S1—O1	123.00 (9)	C2—C3—H3	120.5
O2—S1—N1	110.21 (8)	C5—C4—C3	122.06 (15)
O1—S1—N1	106.70 (7)	C5—C4—N1	119.00 (14)
O2—S1—C7	106.88 (8)	C3—C4—N1	118.94 (14)
O1—S1—C7	105.19 (9)	C4—C5—C6	118.75 (15)
N1—S1—C7	103.02 (8)	C4—C5—H5	120.6
O4—S2—O3	122.60 (8)	C6—C5—H5	120.6
O4—S2—N1	109.14 (8)	C1—C6—C5	119.21 (15)
O3—S2—N1	107.18 (7)	C1—C6—H6	120.4
O4—S2—C8	107.55 (9)	C5—C6—H6	120.4
O3—S2—C8	105.91 (8)	F3—C7—F1	110.51 (18)
N1—S2—C8	102.67 (8)	F3—C7—F2	108.18 (16)
C4—N1—S2	118.56 (10)	F1—C7—F2	109.00 (16)
C4—N1—S1	118.91 (10)	F3—C7—S1	110.98 (13)
S2—N1—S1	122.53 (8)	F1—C7—S1	110.08 (12)
C6—C1—C2	122.17 (15)	F2—C7—S1	108.03 (13)
C6—C1—Cl1	119.27 (13)	F5—C8—F4	110.23 (19)
C2—C1—Cl1	118.56 (13)	F5—C8—F6	109.09 (18)
C1—C2—C3	118.74 (15)	F4—C8—F6	108.97 (16)
C1—C2—H2	120.6	F5—C8—S2	111.24 (13)
C3—C2—H2	120.6	F4—C8—S2	109.37 (13)
C4—C3—C2	119.07 (15)	F6—C8—S2	107.89 (14)
C4—C3—H3	120.5		
			
O4—S2—N1—C4	−155.34 (12)	N1—C4—C5—C6	−179.88 (14)
O3—S2—N1—C4	−20.57 (14)	C2—C1—C6—C5	0.1 (3)
C8—S2—N1—C4	90.75 (13)	Cl1—C1—C6—C5	179.81 (13)
O4—S2—N1—S1	23.61 (12)	C4—C5—C6—C1	−0.4 (2)
O3—S2—N1—S1	158.38 (9)	O2—S1—C7—F3	−40.94 (16)
C8—S2—N1—S1	−90.29 (11)	O1—S1—C7—F3	−173.17 (14)
O2—S1—N1—C4	−151.50 (12)	N1—S1—C7—F3	75.21 (15)
O1—S1—N1—C4	−15.75 (14)	O2—S1—C7—F1	−163.59 (14)
C7—S1—N1—C4	94.75 (13)	O1—S1—C7—F1	64.17 (16)
O2—S1—N1—S2	29.55 (12)	N1—S1—C7—F1	−47.44 (16)
O1—S1—N1—S2	165.30 (9)	O2—S1—C7—F2	77.50 (14)
C7—S1—N1—S2	−84.20 (11)	O1—S1—C7—F2	−54.73 (14)
C6—C1—C2—C3	0.0 (2)	N1—S1—C7—F2	−166.35 (12)
Cl1—C1—C2—C3	−179.66 (12)	O4—S2—C8—F5	−67.64 (18)
C1—C2—C3—C4	0.1 (2)	O3—S2—C8—F5	159.68 (16)
C2—C3—C4—C5	−0.4 (2)	N1—S2—C8—F5	47.42 (18)
C2—C3—C4—N1	−179.97 (14)	O4—S2—C8—F4	170.36 (13)
S2—N1—C4—C5	−102.12 (15)	O3—S2—C8—F4	37.69 (16)
S1—N1—C4—C5	78.89 (17)	N1—S2—C8—F4	−74.58 (15)
S2—N1—C4—C3	77.44 (17)	O4—S2—C8—F6	51.97 (16)
S1—N1—C4—C3	−101.55 (15)	O3—S2—C8—F6	−80.71 (15)
C3—C4—C5—C6	0.6 (2)	N1—S2—C8—F6	167.02 (13)

### Hydrogen-bond geometry (Å, °)

**Table:**

*D*—H···*A*	*D*—H	H···*A*	*D*···*A*	*D*—H···*A*
C3—H3···O3^i^	0.95	2.57	3.496 (2)	164
C5—H5···O1^ii^	0.95	2.59	3.385 (2)	141

Symmetry codes: (i) −*x*+1, −*y*+2, −*z*+1; (ii) −*x*+2, −*y*+2, −*z*+2.
